# Two PerPLEXing Cases of Hashimoto’s Encephalopathy Unresponsive to Steroid and Intravenous Immunoglobulin Therapy

**DOI:** 10.7759/cureus.26853

**Published:** 2022-07-14

**Authors:** Abhinav Karan, Swetha R Nuthulaganti, Yixin Zhang, Fadi Kandah, Maria Gutierrez, Pramod Reddy

**Affiliations:** 1 Internal Medicine, University of Florida College of Medicine – Jacksonville, Jacksonville, USA; 2 Medicine, University of Florida Health Jacksonville, Jacksonville, USA; 3 Internal Medicine, University of Florida College of Medicine, Jacksonville, USA

**Keywords:** encephalopathy with autoimmune thyroid disease, altered mental status evaluation, acute encephalitis, hashimoto’s thyroiditis, hashimoto’s encephalopathy, encephalopathy

## Abstract

Hashimoto’s encephalopathy is a nebulous entity over which much controversy exists. Often referred to as steroid-responsive encephalopathy associated with autoimmune thyroiditis (SREAT), it describes a myriad of neurological sequelae that typically are observed to occur in patients with the presence of thyroid antibodies. We aim to raise clinical awareness of this seldom diagnosed entity as a potential etiology for altered mental status in patients who present with supporting clinical features and elevated thyroid antibodies. While steroid responsiveness is deemed a typical presenting feature of this medical condition, our cases aim to describe two cases that required escalation of therapy to intravenous immunoglobulins, and ultimately, plasmapheresis therapy for improvement in their clinical status. Our patients had a dramatic improvement in their mentation within three to four sessions of plasmapheresis, improving rapidly toward their baseline. Such a dramatic improvement, coupled with the corresponding reduction in their thyroid antibody titer supports the diagnosis of Hashimoto's encephalopathy and highlights the importance of having a low clinical threshold for the diagnosis of this entity in patients who, despite extensive evaluation, reveal no apparent cause for their altered mental status.

## Introduction

Hashimoto’s encephalopathy (HE) is a nebulous entity over which much controversy exists regarding diagnosis and management. Often referred to as steroid-responsive encephalopathy associated with autoimmune thyroiditis (SREAT), it describes a myriad of neurological sequelae that typically are observed to occur in patients with the presence of thyroid antibodies [1]. A hallmark of this disease process, as the name implies, is its response to steroid therapy. We report two cases of HE where the patient’s course showed no improvement with high-dose corticosteroid therapy and subsequent intravenous immunoglobulins (IVIGs), alongside a review of the literature.

## Case presentation

Case 1

A 42-year-old female with a past medical history of type 2 diabetes mellitus, hypertension, and hypothyroidism following total thyroid resection for papillary thyroid cancer presented with an altered mental status. She was in her normal state of health, and had breakfast in the morning prior to the presentation but was later found comatose at noon, with an initial GCS of 4. In transit to the emergency department, she maintained 100% oxygen saturations, however, GCS remained at 4. She was electively intubated in the ED for airway protection and was admitted to the intensive care unit. An initial evaluation for acute encephalopathy revealed a normal computed tomography (CT) of the head. Infectious workup with blood cultures, urinary cultures, syphilis, hepatitis C and HIV testing, and a urine toxicology screen were all negative. She revealed no leukocytosis or metabolic derangements on a complete blood count (CBC) and complete metabolic panel (CMP), and her thyroid-stimulating hormone was only mildly elevated at 5.04 MIU/L (reference range: 0.27-4.2 MIU/L), with a normal free T4 at 1.00 ng/dL (reference range: 0.80-1.73 ng/dL). Inflammatory markers were significantly elevated, with an erythrocyte sedimentation rate (ESR) of >136 mm/hr (reference range: 0-20 mL/hr), and high-sensitivity C-reactive protein of 81.20 mg/L (reference range: 0-5 mg/L). 

**Figure 1:**
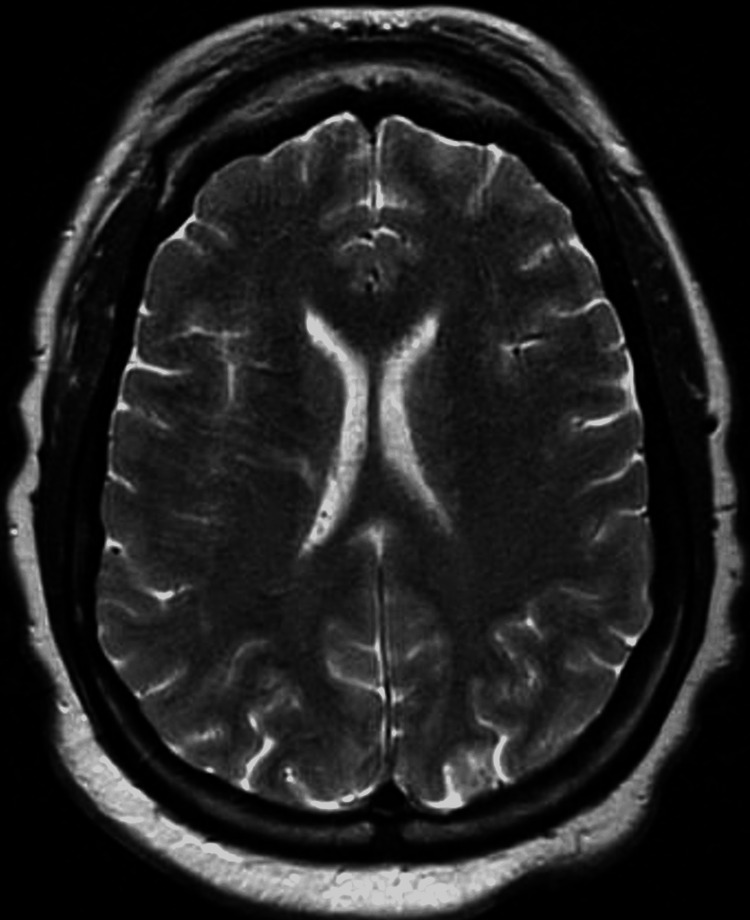
T2 magnetic resonance imaging brain showing grossly unremarkable intracranial findings.

Given the unknown etiology of her acute encephalopathy, further neurologic workup was pursued. A lumbar puncture revealed an elevated opening pressure of 40mmHg and a mildly elevated protein CSF concentration, however, revealed no evidence of underlying infection or oligoclonal banding. CSF analysis for autoimmune encephalopathy was performed with ENC-2 and PAVAL autoimmune screening. Similarly, both magnetic resonance imaging (MRI), MR venography (MRV), and MR angiography (MRA) of the brain were grossly unremarkable (Figure 1). Continuous electroencephalogram (cEEG) revealed diffuse, generalized slowing, consistent with a moderate nonspecific encephalopathy, with no evidence of any epileptiform discharges.

Eventually, the patient was successfully extubated, however, her persistent encephalopathy still prevailed. Further workup with a systemic autoimmune screen, including ANA, rheumatoid factor, anti-CCP, p-ANCA, and c-ANCA was done to rule out any cause of autoimmune cerebritis, however, were all negative. A deep skin biopsy and bone marrow biopsy revealed no underlying intravascular lymphoma or evidence of any vasculitis. The patient was initially treated with a course of empiric high-dose corticosteroids at 1 g daily for five days, however, with no improvement in mental status. Subsequently, 40 g of IVIG therapy for five days was also attempted with futility.

The patient had a prolonged hospital course, remaining encephalopathic for three months following presentation, and dependent on nasogastric feeding. Her inflammatory markers remained persistently elevated, with her ESR at >136 mL/hr. Her CSF autoimmune panel at this point also returned negative. At this point, Hashimoto's encephalopathy was considered. Serum thyroid antibodies were obtained, revealing normal titers of thyroid peroxidase antibodies but significantly elevated anti-thyroglobulin antibody 154.1 IU/mL (reference range: 0.0-0.9 IU/mL).

After a prolonged course and multidisciplinary discussion across specialties, a decision was made to attempt alternate day plasmapheresis (PLEX) therapy for 10 sessions. The patient demonstrated dramatic improvement with PLEX, improving significantly with each session and becoming alert and oriented by her second session. Repeat anti-thyroglobulin antibody testing following completion of PLEX revealed significant improvement. The patient was able to tolerate an oral diet and was conversant and responsive to all commands. She was successfully discharged home and is doing well on outpatient surveillance on a course of an oral prednisone taper for three months. She has had no recurrence during follow-up for the past two years.

Case 2

An 18-year-old male with a history of cerebral palsy presented to the hospital with a generalized tonic-clonic seizure. His mother, who was also the primary caregiver, stated that at baseline the patient is able to conduct a normal conversation and perform small tasks, such as navigating a powered wheelchair or feeding himself, without any difficulties. He was initially successfully managed with intravenous lorazepam and midazolam. After the termination of his seizure, the patient remained encephalopathic with catatonic postures. He was unable to follow any commands or speak meaningful sentences. Initially, a concern was raised for malignant catatonia, but evaluation by the psychiatry team excluded the possibility. He revealed no leukocytosis, anemia, or metabolic derangements, and a normal TSH and free T4.

cEEG monitoring revealed diffuse, generalized slowing consistent with a moderate encephalopathy. Neuroimaging with CT Head was unremarkable, and a lumbar puncture was subsequently performed with only a minor elevation in CSF protein, but otherwise unremarkable, with no signs of infection or oligoclonal banding. An MRI brain could not be obtained due to the presence of deep brain stimulation pads. Given the nonspecific encephalopathy, a concern was raised for possible autoimmune encephalitis. However, a complete serum and CSF autoimmune panel were negative. At this point, we warranted exclusion of Hashimoto’s thyroiditis, and serum thyroid antibodies were collected, revealing an elevated thyroid peroxidase antibody level of 80 IU/mL (reference range: 0-26 IU/mL), and an elevated thyroglobulin antibody of 22 IU/mL (reference range 0-0.9IU/mL).

Given the persistent encephalopathy and unexplained elevation in antithyroid antibodies, a decision was made to treat the patient for SREAT, or Hashimoto’s thyroiditis. Despite a five-day course of 1 g intravenous methylprednisolone, and subsequent IVIG therapy at 40 g daily for five days, the patient had no improvement in his clinical status. The decision was made to proceed with 10 sessions of PLEX therapy. Soon after PLEX started, the patient's mentation improved drastically, and progressively became more alert and less catatonic by the fourth PLEX session. He improved from a state of complete inattentiveness and disorientation to time, place, and person to being alert and oriented to time, place, and person, conversant with his mother and at his functional baseline. Eventually, he tolerated all 10 sessions and was successfully discharged with a prednisone taper and no recurrence of his presentation since discharge.

These two cases serve to highlight two individual cases of Hashimoto's encephalopathy, alongside an emphasis on its varied presentation and in our case, a non-response to steroid therapy. Both cases exemplified a significant increase in the patient's thyroid antibody titer, with a negative CSF autoimmune panel and significant elevation in inflammatory markers (Table 1).

**Table 1:** Comparing relevant inflammatory, endocrine, and cerebrospinal studies between Case 1 and Case 2 TSH: Thyroid-Stimulating Hormone

	Case 1	Case 2
TSH (MIU/L)	5.04	3.23
Free T4 (ng/dL)	1.00	1.23
Thyroglobulin Antibody (IU/mL)	154.1	22.0
Thyroid peroxidase Antibody (IU/mL)	0.7	80.0
C-Reactive Protein (mg/L)	81.2	76.4
Erythrocyte Sedimentation Rate (mm/hr)	>136.0	>136.0
CSF Autoimmune Panel	Negative	Negative

## Discussion

HE is a very rarely encountered disease that is considered distinct from Hashimoto's thyroiditis. The relationship between HE and Hashimoto's thyroiditis is controversial, and although the two may often concurrently be seen in patients, there is much debate as to whether a true relationship exists with the presence of elevated thyroid antibodies [1], with some physicians referring to the encephalopathy as SREAT. It has an incidence estimated at roughly 2.1 cases per 100,000 [1], and very few cases of HE are documented in the literature [2,3].

The clinical presentation of HE is heterogeneous, with a variable course and a broad range of possible presentations. While many patients present with gradually progressing cognitive impairment, dementia, hallucinations, and psychiatric disturbances, approximately 15% of patients have a fulminant course, with coma at the time of presentation [4], as with both patients. It is likely that the diagnosis is underrecognized in clinical practice due to the heterogeneity of its presentation. Compounding this, there currently exists no definitive diagnostic study for HE, with the majority of neurological studies including a lumbar puncture, cEEG, and brain MRI revealing only nonspecific findings [5]. This echoes our own patients, who underwent a similar extensive workup which was largely unrevealing. The relationship between thyroid function and symptoms of encephalopathy is also essential to elucidate, with systematic reviews of a limited number of cases suggesting that 45%-55% of patients present with a hypothyroid profile, 7% with a hyperthyroid profile, and the remaining in a euthyroid state [6].

From case reports of HE, it appears that the vast majority of cases have two key findings that lead to a corresponding diagnosis: both an elevation in thyroid antibodies, and a positive response to corticosteroid therapy [2]. Although our first patient had negative thyroid peroxidase antibodies, the significant elevation in her thyroglobulin antibody titer alongside her encephalopathy led to the consideration of HE as the underlying diagnosis. It is important to recognize that despite an elevation in thyroid antibodies being possible in HE, approximately 15-20% of the population may have positive thyroid antibody titers and should not reliably be used in diagnosis. A recommended threshold of five times the upper limit of normal has been suggested in clinical practice to suggest the presence of HE, but even patients with a smaller proportion of thyroid antibody elevation may respond to a steroid challenge. Similarly, it has also been suggested that some patients with negative thyroid antibody titers have been shown to have positive thyroid antibody titers in the CSF, suggesting some degree of intrathecal production [7]. Therefore, empiric therapy for HE can be considered based on high clinical suspicion even if initial thyroid antibody testing is initially negative since purely intrathecal production of thyroid antibodies with no systemic detection is possible.

Once diagnosed, a positive response to steroids appears to be a pathognomonic feature of HE, with upwards of 98% of patients diagnosed demonstrating improvement in their symptoms [2]. Our patients, however, despite receiving a course of high-dose corticosteroid therapy, remained encephalopathic and required deliberation on further immunomodulating therapies. IVIG, similarly, did not improve either patient's encephalopathy. PLEX has been shown to improve several patients in whom steroids and IVIG have had no benefit for encephalopathies. The data behind plasmapheresis and treatment of HE are lacking, but our patients demonstrated a dramatic improvement in their encephalopathy with PLEX. This response is atypical for HE given the vast majority of cases that are steroid responsive. While our patient’s repeat thyroid antibody titer was undetectable, the utility of surveillance thyroid antibody testing is unknown. It is also essential to highlight the importance of not delaying care while awaiting the results of testing, particularly CSF autoimmune testing, given that our patient had a prolonged course including awaiting the results of the above. While complete or partial neurological resolution occurs in many patients, some studies show a recurrence rate of approximately 16%-25% [2]. Therefore, there is an unclear utility of long-term immunosuppressive therapy to suppress recurrences of HE. Currently, there exist no randomized controlled trials assessing the benefit of long-term immunosuppression for this patient population and we believe this serves as an area for further research into this patient population.

## Conclusions

These cases serve to demonstrate the vast heterogeneity of the presentation of HE. While much controversy exists on its diagnosis, our patients were unresponsive to both steroids and IVIG therapy and required PLEX. Further randomized controlled trials are necessary for the determination of the optimal treatment of this nebulous entity, but this report serves to highlight that plasmapheresis may be an option in certain patients.
